# Volumetrical Characterization of Sheet Molding Compounds

**DOI:** 10.3390/ma3125083

**Published:** 2010-12-01

**Authors:** Alfredo Calvimontes, Karina Grundke, Anett Müller

**Affiliations:** Leibniz-Institute of Polymer Research Dresden, Hohe Strasse 6, 01069 Dresden, Germany; E-Mails: grundke@ipfdd.de (K.G.); mueller-anett@ipfdd.de (A.M.)

**Keywords:** molding compounds, SMC, surface properties, surface morphology, topographic characterization, volumetrical characterization

## Abstract

For a comprehensive study of Sheet Molding Compound (SMC) surfaces, topographical data obtained by chromatic confocal imaging were submitted systematically for the development of a profile model to understand the formation of cavities on the surface. In order to qualify SMC surfaces and to predict their coatability, a characterization of cavities is applied. To quantify the effect of surface modification treatments, a new parameter (Surface Relative Smooth) is presented, applied and probed. The parameter proposed can be used for any surface modification of any solid material.

## 1. Introduction

Due to the topographic heterogeneities of Sheet Molding Compound (SMC) surfaces it is difficult to fulfill optical requirements of coated SMC surfaces for technical applications, especially for the automotive industry. In a previous work [1], it has been demonstrated that the prepreg composition, molding and post-molding conditions define the resulting topography of the surface by controlling its morphology on different length scales. In the present work, morphology was characterized by using the topographic parameters mentioned in [1].

Cavities (pores) on SMC surfaces are normally considered to retain air and produce failures (bubbles) after coating. From a topographic point of view, a ‘coatable’ SMC surface should be isotropic, should have no spikes and should have almost the same-sized, not too deep, regularly distributed micro-cavities.

In order to quantify the surface coatability, additional parameters can be considered: fractal dimension [2], count of cavities, cavity-area average, cavity-volume average, total area of cavities, total volume of cavities and cavities histograms. However, attention to all the mentioned topographic parameters to quantify the coatability is very difficult because the resulting multivariable system is highly complex.

In the present work, we suggest that the quantification of the surface quality by using only one or two parameters might be best. However, this approach requires a better understanding of the mechanism of surface formation. For this intention, a very simple 2D structure model was developed.

Additionally, to quantify the effect of the topographic modification of materials, a new parameter called Surface Relative Smooth (SRS) is presented and applied to different types of surface treatments of SMC materials.

## 2. Materials

Samples of SMC were produced under different conditions of temperature (148 °C), different pressure (3.5 to 14 MPa) and molding time (60 to 360 s) conditions, all of them having a glass fiber content of 10% (formulation B in [1]).

## 3. Experimental Section

### 3.1. Characterization of the topography

A MicroGlider^®^ (FRT, Germany) imaging instrument was used for the optical analysis of the topography of SMC surfaces. Unlike conventional microscopy, which simultaneously images all the points in the field of view and captures a 2D image, the chromatic confocal microscope records only one object point per given unit of time. The field measured is reconstructed by *x-y* scanning. This novel optoelectronic setup, based on a quasi confocal, z-axis extended field, was developed for a high resolution non-contact 3D surface metrology, including roughness characterization and surface flaw detection.

The instrument uses a chromatic white-light sensor (CWL), which is based on the principle of chromatic aberration of light [1,3,4].

Depending on the SMC surface characteristics, other non-contact measuring methods such as scanning electron microscopy (SEM), confocal laser scanning optical microscopy (CLSM), confocal scanning optical microscopy (CSOM), conoscopic holography (CSL), *etc*., can be used. In order to obtain statistically representative topographical data, a proper combination of cut-off length, z-range and resolution has to be used. It is important to note that a method with a very high resolution can be inadequate if the available cut-off length or z-range is too small. On the other hand, the use of a very high resolution and larger cut-off lengths (scan areas) result in a high amount of data and extremely long calculation times, which require a special or non-existent hardware and software.

All topographic measurements were done using the optimal sampling conditions, cut-off length and lateral resolution, obtained according to the method presented in [1]. By using this method, sample size (cut-off length) and sampling frequency (lateral resolution) were obtained as 6 mm and 5 µm respectively. In other words, using these sampling conditions, each topographical map, *i.e*., ‘sample’, contains 1.44 million coordinates; therefore, topographical parameters practically do not depend on measuring position on the SMC plate. In all cases, standard deviations were smaller than 1%. To avoid the effect of mold topography on the resulting SMC surface, the same mold position was studied for all samples [1,7].

### 3.2. Surface modifications

Three different modifications of the SMC surfaces were applied: (a) mechanical cleaning by cloth using ethanol-benzene solution (50–50% in volume), (b) powerwash (50 °C, 3 bar, 2 mins, 1% ESKAPHOR N 6502 B), and (c) cleaning by CO_2_ pressure (2 bar) for different durations.

The aim of the present work is to propose a new volumetrical parameter to the study of topographical changes on SMC surfaces and to show its potential in quantifying the effect of three representative industrial treatments. A deeper analysis of the physical or chemical mechanisms of these treatments is beyond the scope of the present work.

## 4. Results and Discussion

### 4.1. A profile model to understand the formation of cavities on SMC surfaces

Though SMC surfaces are not periodic surfaces, *i.e*., they do not show any repeating units, some periodicity due to regular polish of the metallic mold permits the use of a periodic profile model in order to understand the formation of cavities as a result of the combination of the mold irregularities with molding and post-molding conditions.

**Figure 1:**
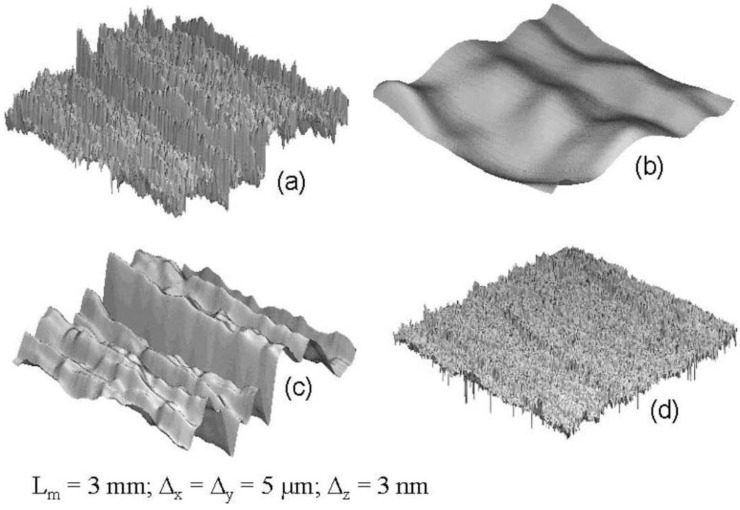
Total Sheet Molding Compound (SMC) topography (**a**), isolated short waviness (**b**), isolated mean roughness (**c**) and isolated arithmetic mean roughness (**d**). L_m_ is the cut-off length. ∆_x_ and ∆_y_ are the lateral resolutions and ∆_z_ is the vertical resolution.

**Figure 2:**
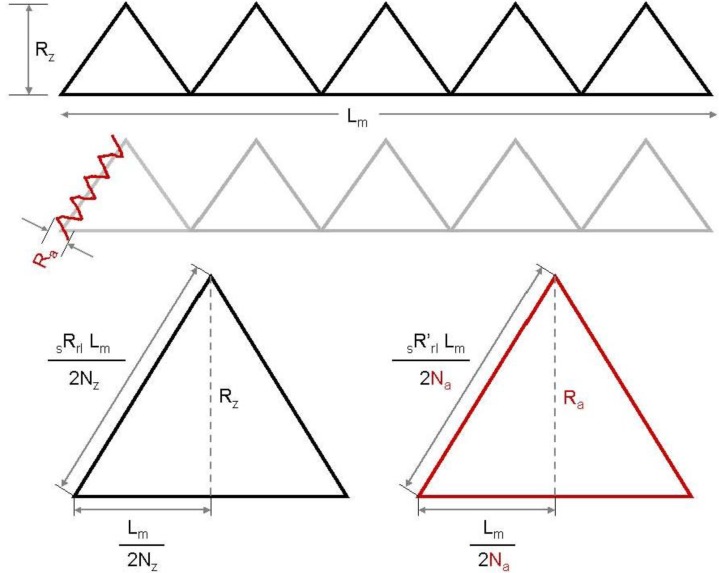
2D profile using triangular waves to describe the formation of long and short waves and their relation with R_z_ and R_a_.

The following approach to develop a structural model does not take into account the short waviness; only mean roughness and arithmetic roughness are considered to suit the total SMC topography (Figure 1). Modeled profiles consist of triangular periodical waves of two length scales: the first scale describes the mean roughness R_z_ and the second one the arithmetic mean roughness R_a_ filtered by Fast Fourier Transformation [5], as shown in Figure 2. Using calculated values of surface Wenzel roughness factor (r), defined as the quotient *real area/projected area* [6], the number of long waves (N_z_) and short waves (N_a_) can be obtained:

(1) $$N_{z}=\;\frac{L_{m}}{2R_{z}}\sqrt{{r_{z}}^{2}\;-\;1}$$

(2) $$N_{a}=\;\frac{L_{m}}{2R_{a}}\sqrt{{r_{a}}^{2}\;-\;1}$$

**Figure 3:**
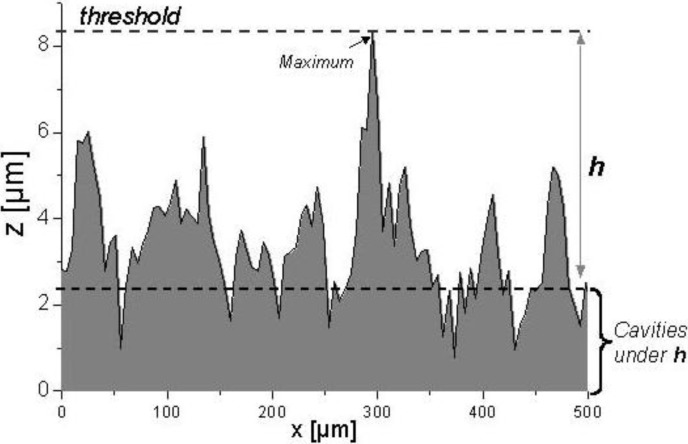
Quantification of cavities by ‘filling quantity operation’ [7] under 6 µm of highest point.

The quantity of cavities and their mean area were calculated by applying ‘filling quantity operations’ [7], with a threshold value (maximum z-height) of 6 µm, measured under the highest point of the surface after filtration of short waviness (Figure 3).

The threshold is an arbitrary value that depends on surface morphology. The purpose is to take a reference plane that can serve as “failure”- limit for all the surfaces to be studied. As it will be shown later, the volumetric characterization of SMC surfaces produced by another better polished metallic mold demands the use of a threshold of only 2 µm.

**Figure 4:**
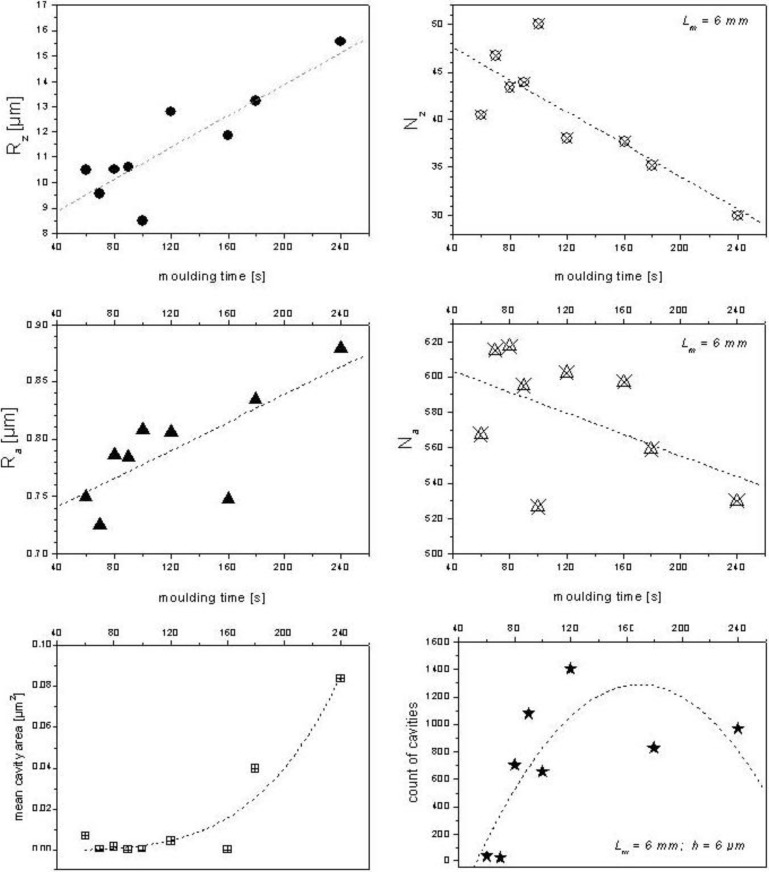
Measured values (R_z_, R_a_, mean cavity area and count of cavities) and calculated N_z_ and N_a_ as a function of molding time for a SMC-1 surface.

**Figure 5:**
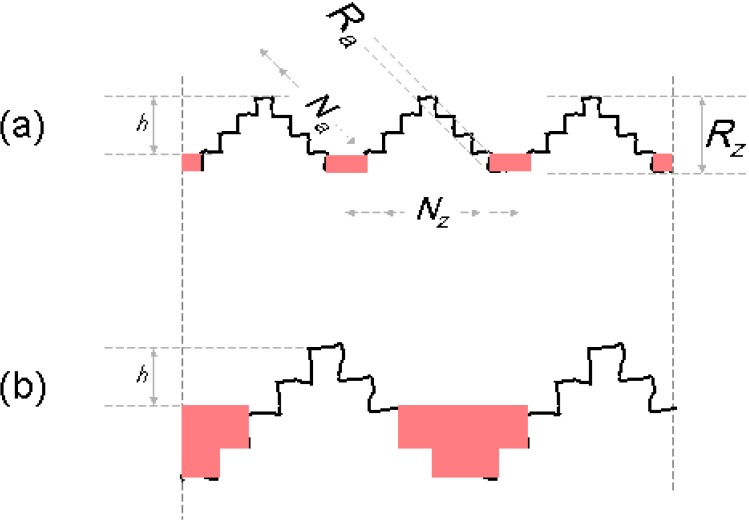
Modeled SMC-1 profile before molding (**a**), schematization of predicted profile after 240 s of molding (**b**). Sizes are not scaled.

Applying the model for a surface of SMC-1 (formulation B in [1]) manufactured by a pressure of 5 MPa, the resulting quantity of cavities and their cross-sectional mean area (Figure 4) could be predicted schematically by the behavior of R_z_, N_z_, R_a_ and N_a_ as functions of molding time (see Figure 5). In this case, molding time increases R_z_ and R_a_. At the same time, long and short waves decrease their number. The process results in an increase of cavity cross-sectional area (as a mean value), which is predicted by the conceptual model described above. However, a decrease of cavity count, predicted by the model, occurs only in the last 120 seconds of molding.

**Figure 6:**
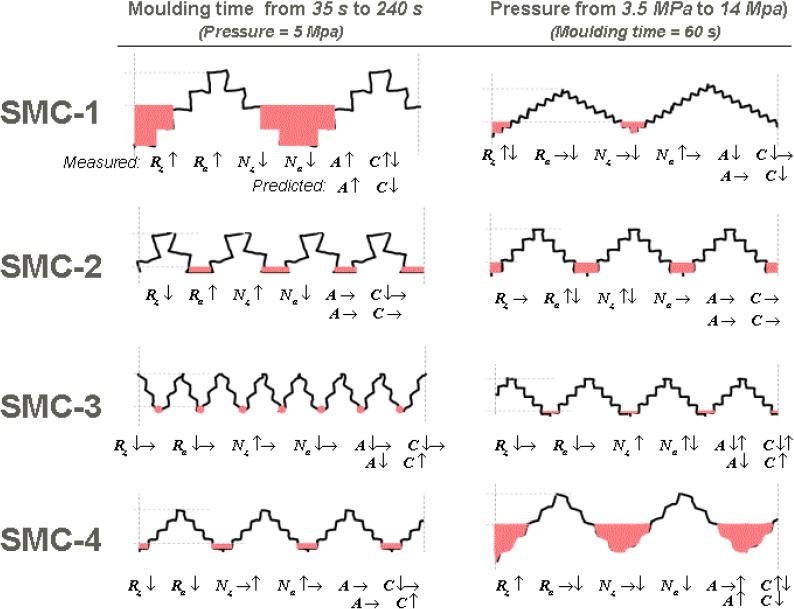
A qualitative comparison between measured and predicted parameters. A: mean cavity area, C: count of cavities. ↑ denotes increase, ↓ denotes decrease, → denotes no change.

The same analysis, applied to other SMC formulations, having different compositions of filler and additives mentioned in [1], for different molding time and pressure values, shows that the model can conceptually predict the morphological modification of the profiles (Figure 6). In the case of SMC-3 at different molding times, the effect of short waviness (which is very important according to [1]), does not allow the successful application of the model. The best prediction was obtained for SMC-2, for which measured waviness (W_z_) is the smallest of those investigated.

### 4.2. Characterization of cavities and prediction of coatability

**Figure 7:**
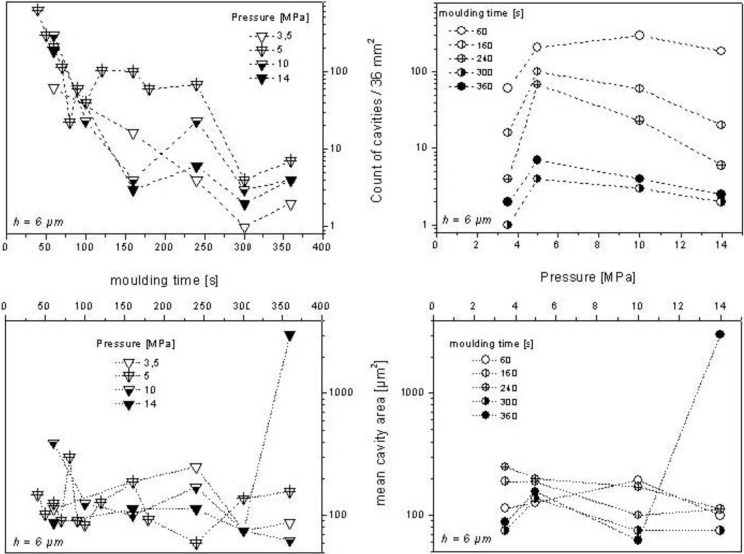
Count of cavities (top) and mean cavity area (bottom) under 6 µm from the highest point by different pressures (right panels) and molding durations (left panels) for SMC-2 (Figure 6) surfaces. Samples studied (36 mm^2^, L_m_ = 6 mm) correspond to the same position of metallic mold.

The number of cavities under defined threshold and their mean area in a defined sample area (*L_m_* × *L_m_*) can be used to compare and quantify the coatability of SMC surfaces. By using both parameters, the frequency and size of structural failures can be quantified. Figure 7 shows the quantification of cavities in order to characterize the coatability of a SMC-2 surface. As explained in Section 3.1, the statistical reliability of the topographical data was guaranteed by the use of the optimal sampling conditions [1]. The overlapping behavior observed in Figure 7 is a consequence of the prepreg placement procedure and adhesion between mold and SMC surface during detaching [1,7].

According to this characterization method, at a pressure of 3.5 MPa and a molding time of 300 s the best coatable surface is formed. Only one cavity was found under a threshold of 6 µm, whose area is about 75 µm^2^. Lower pressure values lad to lower adhesion during the separation of the SMC surface from metallic mold; as a consequence, the resulting topography is more regular.

At the highest pressure (14 MPa), only two cavities were found after 300 s of molding and four cavities after 360 s. However, by this duration of molding, mean area of the cavities increased dramatically from 75 µm^2^ to 3000 µm^2^.

### 4.3. Surface Relative Smooth parameter (SRS) to characterize the topographic modification

**Figure 8:**
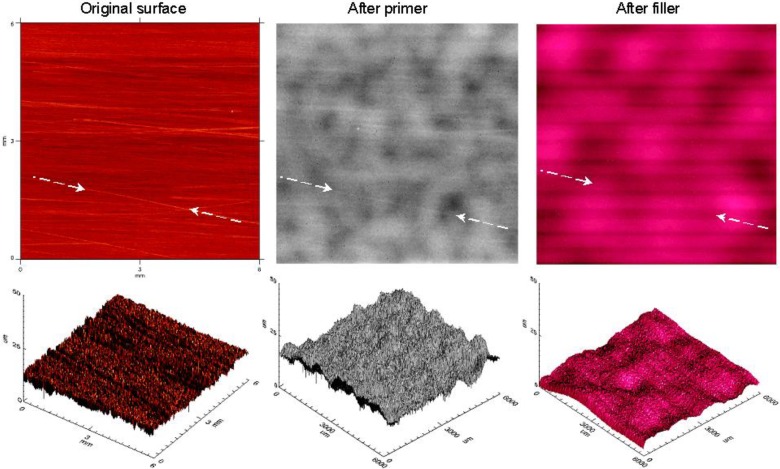
Peaks marked by arrows, produced by mold irregularities, remain after application of primer and, occasionally, after filler.

By using the count of cavities and their mean area as characterization parameters, only deep points are considered as potential causes of surface failures after coating by formation of undesired bubbles. However, tests demonstrate that grains (peaks), which are normally produced by deeper regions on the metallic mold, could not be successfully coated. Figure 8 shows that after the first two steps of the coating process, primer (B1402 + B1426 Wörwag) and filler (7245 Anthrazit Hammelrath), some grooves remain at the surface.

**Figure 9:**
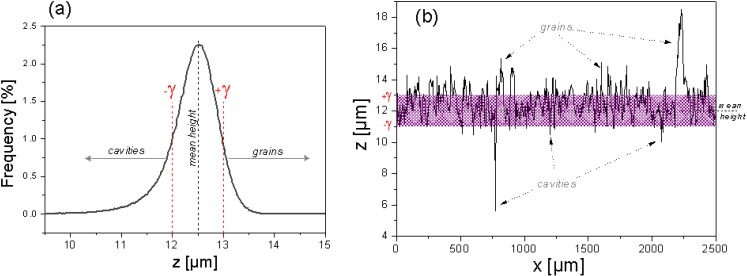
Thresholds +γ and –γ define cavities and grains in a heights histogram (**a**), or cavities and grains in a surface profile (**b**).

To quantify the cavities as well as the grains of SMC surfaces, a critical parameter is needed. Taking the statistical mean height as a reference height value, it is possible to calculate the quantity and volume of all irregularities above a positive threshold (+γ) and under a negative threshold (-γ), according to Figure 9. Knowing the total cavities volume (VC) and the total grains volume (VG), it is possible to characterize quantitatively the topographic modification of a surface by any treatment using the Surface Relative Smooth parameter (SRS) defined by the equation:

(3) $$SRS=1-\frac{V_{Cf}+V_{Gf}}{V_{Ci}+V_{Gi}}$$

where i and f denote before and after topographic modification, respectively. For a practical application of this parameter, it is recommended to use percentages. In this case, a SRS value of zero denotes no topographical changes and 100% means a total surface smoothing (elimination of all cavities and grains). Negative values of SRS mean the formation of a more rugose surface after treatment.

**Figure 10:**
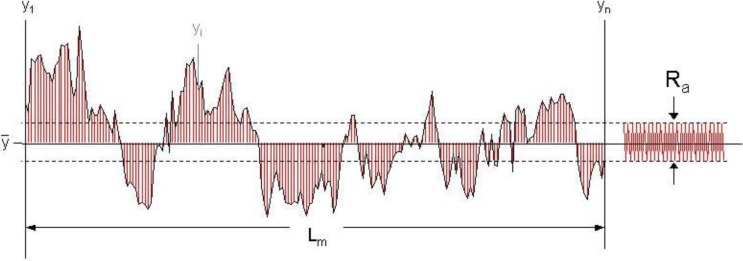
Arithmetic mean roughness R_a_ of a profile.

When SRS parameters are used it is highly recommended to compare them in the same sample area. The selection of a value for the threshold γ depends on surface topography; as a first approach ½ R_a_ can be acceptable. In this case, all cavities and grains are formed by points located above or below the R_a_ amplitudes, as shown in Figure 10. The most important condition is that γ must allow topographic differences before and after treatment to be perceived and must be constant during the topographic characterization. Comparison between SRS values only makes sense if the γ value used is the same.

**Figure 11:**
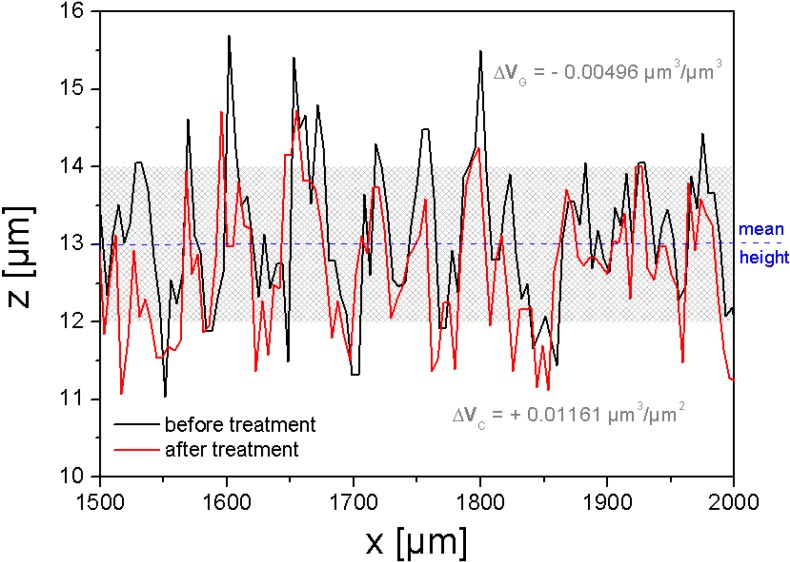
Modification of a SMC surface by cleaning with benzene-ethanol. Both profiles correspond to the same coordinates.

SRS is very sensitive to small changes in topography. For example, after mechanical cleaning (rubbing with cloth) of a SMC surface by a mixture of benzene and ethanol (50%-50% in volume) the topography changes. Figure 11 shows the profile modification measured over the sample SMC-2, maintaining the measure coordinates.

**Figure 12:**
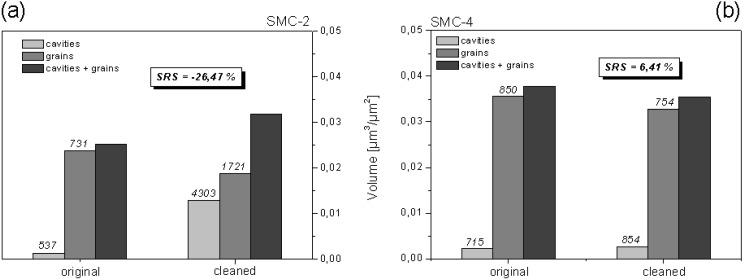
Complete characterization and comparison of modification by cleaning with benzene-ethanol between SMC-2 and SCM-4 surfaces. Evaluated area was 30.6 mm^2^. γ = 1 µm. Values above bars denote cavities and grains counted.

A complete analysis by SRS should also consider an increase or decrease of counted cavities and grains. Figure 12 shows the values before and after a surface treatment of a fixed area of 30.6 mm^2^.

In the case of SMC-2 (Figure 12a), results of the volumetric characterization indicate that solvent mixture removes monomer residues from deeper points, resulting in more and larger cavities. For the same reason, solvents increase the number of grains while friction during mechanical cleaning reduces their total volume by wear. The effect of solvents prevail on the effect of mechanical wear resulting in a more rugose surface (SRS = –26.47%). On the contrary, SMC-4 is more resistant to solvents as can be seen in Figure 12b. During treatment, mechanical wear removes grains in number and volume, leading to a smoother surface (SRS = 6.41%).

**Figure 13:**
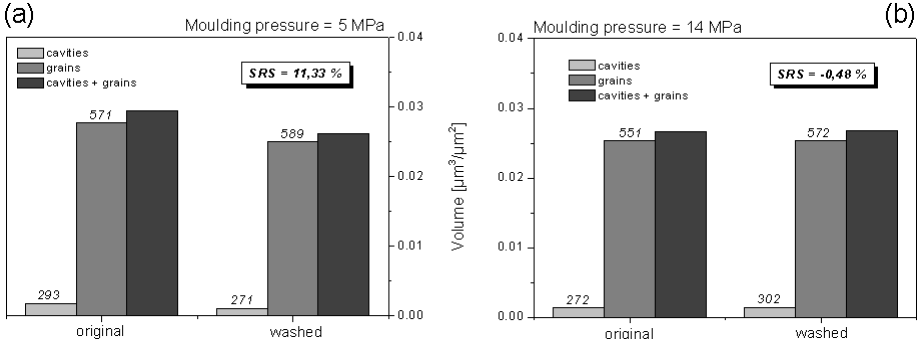
Complete characterization and comparison of a modification by powerwash. Both surfaces were molded for 160 s, but under two different pressures. Evaluated area was 36 mm^2^. γ = 1 µm. Values above bars denote cavities and grains counted.

The effect of molding conditions on the quality of a surface could be characterized by SRS. Figure 13 shows the volumetrical characterization of a SMC-2 sample produced by 5 MPa and 14 MPa of molding pressure after treatment by powerwash (water pressure washing, 50 °C, 3 bar, 2 min, 1% ESKAPHOR N 6502). According to these results, SMC-2 molded under higher pressure (14 MPa) (Figure 13b) seems to be more resistant to powerwash treatment, because almost no change in SRS value was observed (SRS = −0.48%).

**Figure 14:**
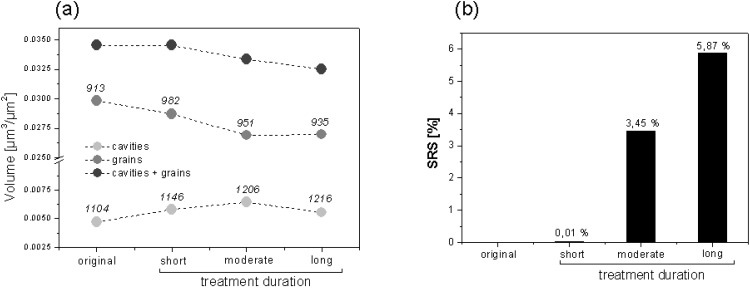
Sequential smoothing by CO_2_ pressure applied on a SMC-4 surface molded for 160 s at 5 MPa.

Besides the use of SRS values to compare the behavior of different surfaces under defined type of treatment, the parameter can be used to compare the effectiveness of different treatments on a defined surface. Figure 14 shows the effect of different treatment intensities (by quantifying treatment duration) of the same CO_2_ pressure (2 bar) applied over SMC-4 surfaces previous molded for 160 s under 5 MPa of pressure. The results indicate that the treatment improves smoothing of the surface depending on its duration. CO_2_ pressure increases the number of cavities and its volume, but at the same time compresses the grains volume, which controls the smoothing of the surface. Values of cavity and grain volumes after long-term treatment do not follow the same behavior because the mean height position is modified to a lower plane after two sequential treatments. However, the total volume (cavities and grains) describes volumetrical changes occurred.

SRS values should be always accompanied by cavities and grains count information in order to suitably evaluate the topographical changes produced by the modification.

**Table 1:** Traditional topographic characterization using R_a_ compared with SRS method.

**Treatment**	**Process**	**Surface**	**Conditions**	R_ai_	R_af_	**R_af_−R_ai_**	**SRS**
				*μm*			
**cleaning**	Benzene-Ethanol	SMC-2	cloth	0.736	0.852	**0.1160**	**−26.47%**
		SMC-4		0.737	0.735	**−0.0020**	**6.41%**
**washing**	Power-wash	SMC-2	5 MPa	0.728	0.728	**0.0000**	**11.33%**
			14 MPa	0.725	0.730	**0.0050**	**−0.48%**
**cleaning**	CO_2_ pressure	SMC-4	No treatment	0.808	-	**-**	**-**
			short	0.808	0.869	**0.0610**	**0.01%**
			middle	0.808	0.863	**0.0550**	**3.45%**
			long	0.808	0.823	**0.0150**	**5.87%**

## 4. Conclusions

By comparing SRS values measured with a traditional topographic parameter such as arithmetic mean roughness, the effectiveness of the presented characterization method is evident. According to Table 1, the information provided by measured values of R_a_ before and after treatment and the difference (R_a*f*_–R_a*i*_) are insufficient to describe all the occurred topographic transformations.

In the proposed method, the SRS parameter can be complemented also with the use of cavity and grain counts, as well as their volumes in order to provide a complete view of topographical changes due to surface treatments.

Through the inspection of the magnitude of cavity volume and grain volume, it is clear that the former dominates. This fact does not depend on the threshold g used for the calculation, but on the mean height of the topographical data. This confirms the statistical validity of SRS parameter.

The parameter proposed can be also used for any surface modification of any solid material.
